# Research on the Construction of College Football Classroom Practice Teaching System Model Based on Big Data Analysis

**DOI:** 10.1155/2022/5018033

**Published:** 2022-02-14

**Authors:** Bo Zhang, Wei Ren

**Affiliations:** ^1^PE Department, North Sichuan Medical College, Nan Chong 63700, China; ^2^PE Department of Public Teaching Centre, Cheng DuMedical College, Cheng Du 610500, China

## Abstract

With the rapid development of information technology, the traditional single classroom teaching and passive learning methods of students can no longer meet the needs of all-round development of college students, and its urgent need to integrate with information technology. This article is aimed at the problem of lagging feedback on training results in the traditional teaching model, teachers' active control, students' passive obedience, ignoring the development of students' personality in college football classrooms, and the inability to carry out personalized tracking and quantitative improvement of the training process of students' related abilities. We constructed a college football classroom practice teaching system model based on big data analysis from the perspectives of establishing big data teaching resources, and implementing personalized resource recommendation, optimizing the traditional teaching process, integrating quantitative training, measurement and recording, implementing quantitative intervention, etc. Colleges and universities have carried out experimental observations. Through continuous observation and comparison, it is found that college football classroom practice teaching under big data is more conducive to improving students' football skills and theoretical level than traditional teaching. This model makes full use of the advantages of big data and the combination of practical teaching methods, which can bring students a different learning experience and obtain good teaching effects. It has guiding and reference significance for college football practical teaching.

## 1. Introduction

In recent years, the field of physical education has made great progress relying on the rapid development of information technology and network technology. However, in the traditional college football classroom teaching evaluation, the results of learning behavior are often obtained through manual recording and measurement, and the efficiency of data record collection, analysis, and graphic and visualization work processing is low; in the teaching form, teachers actively control, and students passively obey, leading to the students' theoretical learning and understanding being not deep enough; in addition, in the practical teaching link, intensive exercises are mainly carried out based on the data results to improve the quality of students' training and lack of attention to the process of training behavior and ignore the different personalities and characteristics of the students during the training process. Big data is a collection of multiple data carriers. Due to its rapid transmission, and rich and diversified data characteristics, it can perform in-depth mining of collected data. It can better provide real-time data trends for college teachers and students in the classroom practice teaching process. This application will play a very important role in college teachers and students' football teaching practice.

In the context of the era of big data, massive amounts of data are gradually being integrated into modern teaching. How to use these data and information fully and efficiently to better serve sports training and teaching and to further improve the teaching mode and process is currently a hot research topic. Is also one of the problems that educators must solve [1].

At present, teaching workers and scholars have proposed some concepts and methods of using big data to optimize the teaching mode and content of college football. Downer [2], Griffin and Murtagh [3] showed that quantitative teaching can significantly improve students' theoretical reading ability; Gallagher [4], Stromgren et al. [5] conducted quantitative teaching experiments in mathematics teaching, and the results showed that quantitative teaching has a significant effect on students who have difficulty in learning football training. Lindsley [6] introduced what is the era of big data and the typical cases of data application in the era of big data and analyzed why classroom teaching in the era of big data changes from the perspectives of teachers and students and how to respond to the changes; Binder [7] teaches APP courses based on big data, through the experimental process of a large number of student self-study effect analysis data to analyze the 3 types of students in the experiment, according to their process learning data to analyze their learning methods, so as to find students' efficient self-study mode; Griffin [3] aimed at the big data trend, carried out targeted classroom teaching evaluations for college football classrooms, realized the diversification of evaluation subjects, combined formative and summative evaluations, and integrated quantitative and qualitative evaluations; Lambe et al. [8] aimed at the big data trends, put forward the concept of big data classroom, analyzed the characteristics of big data classroom, designed the construction plan of big data classroom, and pointed out the connotation and development trend of big data classroom.

## 2. Our Works

This article will launch a research on the construction of the college classroom practice teaching system model in the context of big data. Through the proper use of big data, it can bring greater value to the football teaching classroom, so that colleges and universities can obtain more and more detailed information to the greatest extent. The rich data provides decision-making and new ideas for the innovation of teaching practice in colleges and universities in the future.

## 3. Related Theories

### 3.1. Behavioral Learning Theory

Skinne [9] is one of the founders of American New Behavioral Psychology. He believes that human behavior is mainly an operational behavior composed of operational reflections, and operational behaviors are behaviors that act on the environment to produce results. Almost all human behaviors are the result of operational enhancement. Football practice teaching can further improve and strengthen football skills through accurate feedback of students' football skills through data. In addition, students can change the reactions of others through the influence of reinforcement. In the context of teaching practice, operational behavior is more representative, so operational reflex is particularly important in the learning process. In 1954, Skinne introduced this theory into teaching, thinking that teaching is to put forward the goals that students should achieve and control the learning process, supplemented by training, feedback, and corrective remedies, to form the required behavior that is to achieve the goal and give it immediately. Reinforce those behaviors that deviate from the goal or fail to reach the goal; they are corrected without strengthening [10–14].

### 3.2. Master Learning Theory

The American educational psychologist Bloom put forward the theory of mastering learning for the first time. He believed that mastering learning is to take “all students can learn well” as the guiding ideology, pay attention to the differences between individuals, and adopt various teaching methods to ensure that every student can master the knowledge learned and complete the learning objectives [15–17]. Its basic process is as follows: student orientation-regular teaching-finding mistakes-correcting mistakes-reevaluation.

In the college football classroom practice teaching based on big data analysis, students can learn independently with their own training data and corrective directions and communicate and discuss their doubts with teachers and classmates through the online teaching platform. Teachers can adjust teaching strategies in time through the large amount of data accumulated in the practical teaching process and then gradually complete the teaching goals. This mode is for all students, ensuring that every student can master the knowledge and skills of football theory.

### 3.3. Training and Measurement

The procedural method of football practice teaching in colleges and universities requires students to train daily and measure their learning effects, that is, to spend a certain amount of time every day for football training and measurement. Football training is the basis of measurement, and this process requires long-term development and continuous recording. Generally speaking, the measured frequency data will be recorded by the teacher in a standard variable speed chart. The chart can accurately predict when the student's knowledge or skills will meet the training requirements to determine whether the current student's learning performance has improved significantly over time. Whether the intervention measures need to be modified. In the teaching process, the teacher can collect corresponding data according to the students' daily football training, measurement and recording, and frequently monitoring the student's training behavior status to obtain the student's development [18–21].

## 4. Construction of College Football Practice Teaching System Model Based on Big Data Analysis

**Figure 1:**
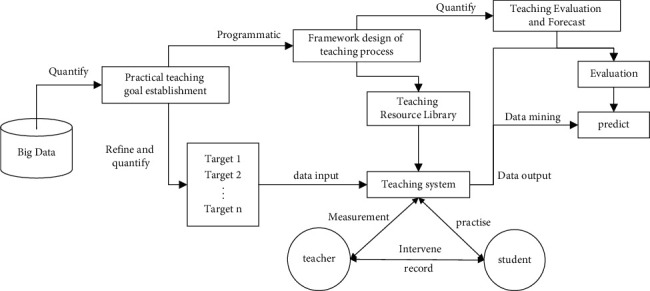
University football classroom practice teaching system model based on big data analysis.

In the traditional teaching environment, teachers are more inclined to apply a certain mature teaching model, and practical teaching under big data analysis is often regarded as a teaching evaluation strategy or a teaching method for a certain course rather than a teaching model. Practical teaching under big data analysis first encountered the obstacles of teachers' thinking and ideas in the application and promotion. Big data breaks through many constraints of the traditional teaching environment and is conducive to encouraging teachers to accept and recognize practical teaching under big data analysis in terms of thinking. It is of great significance to promote the development of practical teaching under big data analysis and to promote the application of practical teaching under big data analysis. For this reason, this research constructs a practical teaching model based on big data analysis based on big data from the three dimensions of teaching goal establishment, teaching process frame design, teaching evaluation, and prediction, as shown in Figure 1.

### 4.1. Establishment of Practical Teaching Goals in Football Classrooms in Colleges and Universities

The clear goal of practical teaching is the logical starting point for the implementation of teaching, and it is also an important basis for testing the success or failure of teaching. Accordingly, the primary task of practical teaching under big data analysis is to establish practical teaching goals. In the traditional teaching environment, the teaching goal can be ambiguous. For example, in the basic computer course, the teaching goal of a certain course can be “proficient in the conversion of decimal and binary,” and the “mastery” is a fuzzy Degree word. However, in practical teaching under big data analysis, quantifiable teaching goals must be designed; that is, there must be a quantitative explanation and description of the degree of knowledge or skills mastered by students; the basic idea of explanation is the decomposition and refinement of the problem. The way of description is quantification. In other words, in practical teaching under big data analysis, each teaching objective should be transformed into a corresponding problem, and each problem should be decomposed and refined into small problems that can be quantified and described. For example, “proficient in the conversion of decimal and binary” can be transformed into “completing 5 decimal and binary exchange problems within 1000 within 3 minutes, and the accuracy rate is 100%”; the “proficient” here has been decomposed and refined, After quantification, it includes not only the accurate mastery of knowledge or skills, but also the speed of applying knowledge or skills, so as to keep fully in line with the measurement indicators of practical teaching under big data analysis.

### 4.2. The Framework Design of Programmatic Teaching Process in College Football Class

Practical teaching under big data analysis originated from Skinne's program teaching, so proceduralization is the core element of practical teaching under big data analysis. Designing a procedural teaching process framework is the key to ensuring the effective implementation of practical teaching under big data analysis. The programmatic teaching process framework designed in this research refers to the process and rules of practical teaching based on big data implementation under big data analysis, including:

#### 4.2.1. Establish a Big Data Teaching Resource Database and Implement Personalized Resource Recommendation

The essence of programmed teaching is an input and output system; that is, input teaching resources and output the results of football training of students. In the traditional teaching environment, due to limited teaching resources and lack of information technology, the input and output system takes the entire teaching class as the basic particle, which cannot guarantee the individualized development of students. In response to this problem, this research proposes to establish a big data education resource library to manage massive digital teaching resources; at the same time, the basic particles of the input and output system are refined from the class to each student, using intelligent recommendation technology, according to the students. The characteristics of learning are equipped with different high-quality teaching resources, and personalized teaching is implemented.

#### 4.2.2. Optimize the Traditional Teaching Process and Incorporate Quantitative Training, Measurement and Recording

This research makes full use of the advantages of big data technology to optimize the traditional teaching process, on this basis, incorporates quantitative training, measurement, and recording, and conducts practical teaching under big data analysis, so as to provide support for the next step of teaching decision-making and learning intervention. Specifically, this research has conducted two types of attempts at the operational level:

  ① Based on excellent online resources to assist teaching, that is, using micro-classes as the content and youtube as the platform, practical teaching under big data analysis can fully meet the teaching needs-first, and students pay attention to the youtube course number with their real names; then, students click on the youtube platform Online micro-class resources, theoretical study, and offline training; finally, the background system automatically records the student's learning behavior to form each student's theoretical learning trajectory and analysis results.
  ② Practical teaching based on project-oriented task-driven, that is, based on the computer basic course training and evaluation system as a platform, in the traditional project-oriented task-driven teaching framework, practical teaching under big data analysis is carried out-first, students log in to the system for practical training Drill, and you can submit every task you complete; otherwise, you cannot enter the next task stage; after the entire project is completed, submit it to the system for scoring; finally, the system records the time and points lost for students logging in and completing each training task in real-time (Error) and the final score form the student's learning trajectory and error problem domain.

#### 4.2.3. Implement Quantitative Interventions

Quantitative intervention is the essence of practical teaching under big data analysis. In a big data environment, whether it is youtube or a computer basic course practice evaluation system, teachers and students can communicate across time and space, and communication records can be traced back and forth. Based on the measured and recorded football training behaviors of students, teachers can judge whether students can successfully achieve the teaching goals—if they can be achieved, there is no problem; if they cannot be achieved, there is a problem, and intervention is required. Specifically, this research carried out targeted interventions at the operational level according to special problems and general problems: for the special problems of individual students, real-time point-to-point intervention and correction are carried out through instant messaging tools; for common problems that are more reflected, they are unified intervention and correction through teaching blogs, youtube, and Moments. Intervention is an iterative task, and football training, measurement, and recording are combined with intervention. In each stage of the training process, the effectiveness of the training is tracked and feedback point-to-point, and it becomes a cyclic iterative process. This cyclic iteration will not stop until all students have reached the theoretical knowledge of football or football skills required by the teaching goal.

### 4.3. Evaluation and Forecast of Practical Teaching in Football Class in Colleges and Universities

In the traditional teaching environment, teaching evaluation may be a vague empirical judgment, such as using degree words such as “excellent,” “good,” “medium,” “pass,” and “poor” to evaluate students' learning performance; or simple Score judgment, such as the final exam score, mid-term exam score, total score, and average score to evaluate the student's learning results. In the big data environment, the fusion application of many advanced technologies such as sensor technology, face recognition technology, and learning analysis technology makes the evaluation of practical teaching under big data analysis from the beginning to the end of the accompanying teaching behavior and can be used to assess what has not happened yet. Quantitative forecasts for the future. For example, Zheng Yiwen et al. proposed a classroom big data collection technology, which integrates student sitting posture measurement system, eye recognition system, and noise recognition system. By acquiring some big data on students' living conditions in the classroom, it can be more accurate Interpretation, analysis, and judgment of students' learning conditions (such as class arrival, concentration of thoughts, classroom activity, and physical fatigue); this technology has high real-time performance, making it possible to implement quantitative and effective attention to each student possible. It can be seen that the practical teaching evaluation based on big data analysis is a kind of real-time evaluation of the whole staff, the whole process, and all directions. In the practical teaching model based on big data analysis, teaching evaluation mainly relies on technical means (including big data collection, educational data mining, learning analysis, and data visualization technology) and is automatically monitored and automatically monitored by various intelligent teaching systems. Analyze the learning situation of students and give real-time feedback to those who need it; teachers, students, parents, etc. can query and generate visual evaluation reports according to their own needs. Prediction refers to the comprehensive analysis of each student's learning performance data and other system data (including various education systems, evaluation systems, and expert systems) to form a data decision support system and conduct a comprehensive analysis of the student's learning performance in the future. Forecast, and then put forward relevant football training improvement suggestions or countermeasures based on the forecast results.

### 4.4. Selection and Evaluation of Experimental Indexes in College Football Class

A large number of practical studies have shown that the use of big data analysis in physical education can better improve students' physical fitness and technical level, cultivate students' sensitivity to training quantitative results, and exercise students' ability to independently develop quantitative results. Compared with the traditional teaching mode, its effect is more significant. Therefore, in order to further study it, to explore whether the application of this model in college football classroom practice teaching can have a positive impact. Based on big data analysis of college football practice teaching methods and combined with previous research results, the following core weight data indicators recorded in the teaching system in real time in the past are selected to carry out experimental research in a college:

#### 4.4.1. Physical Fitness

Physical fitness refers to the strength, speed, endurance, agility, and other functional capabilities of the human body in activities and is the basis for mastering sports techniques and improving sports performance. After quantifying and screening through big data, scoring is performed, and the top three items with the highest weight coefficients are selected as the test indicators of physical fitness in this experiment. After screening, the 5*∗*25-meter retracement run, the 30-meter sprint, and the standing long jump are used as the physical fitness test indicators (see Table 1 for details).

**Figure 2:**
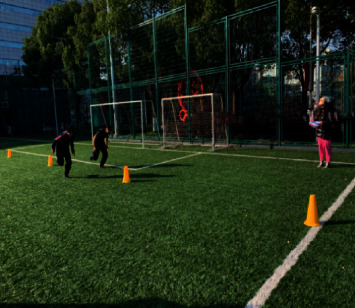
Turn-back running test.

Before and after the experiment, the two groups of students were tested for their physical fitness. The test content included three indicators: 30-meter sprint, standing long jump, and 5*∗*25-meter return run. The 30-meter sprint can reflect the quality of speed, the standing long jump can reflect the explosive power of the lower limbs, and the 5*∗*25-meter return run can reflect the physical quality such as speed, endurance, and agility.  Figure 2 shows the test site, where the experimental group performed a 5*∗*25-meter turnback run.

#### 4.4.2. Football Technology

**Figure 3:**
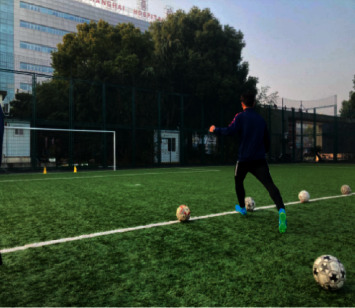
Football technical assessment.

According to the technical assessment requirements of efficient football practice teaching, and consulting football related teachers, determine the frontal bump and shot of the instep as the football technical test indicators. The assessment content and scoring standards are as follows:

  ① Bump the ball on the front of the instep
  Test method: the teacher starts to issue the order after the tester is ready. The tester picks up the ball with his toes or throws the ball with his hands and then uses the front of the instep to make continuous ball bumps. During the ball bumping process, the tester tries his best to turn his feet alternately. The ball can also be adjusted with one foot continuously, and the test is deemed to be terminated once the ball hits the ground. Each person has three test opportunities to get the best result. The scoring standards for the ball-dumping are shown in Table 2:
  ② Shot
  Test method: boys shoot from the penalty area line, girls shoot from the penalty spot, divide the goal into three equals, shoot two points on both sides, and score one point in the middle. Each person has five chances and accumulates points.  Figure 3 shows the test site of the football shooting technique of the experimental group.

#### 4.4.3. Theoretical Assessment

It is proposed to select certain college football theory assessment regulations. There are three sets of theoretical examination papers ABC. One week before the examination, teachers randomly select one of them as the final examination papers. Take the three knowledge modules of football overview, basic football skills and tactics, and football game rules as the theoretical investigation content. It mainly includes four question types: judgment, filling in the blanks, noun explanation, and question and answer. There are 22 questions in total, with a total score of 100.

## 5. Experimental Results and Analysis

### 5.1. The Results and Analysis of Various Indicators before the Experiment in the Experimental Group and the Control Group

#### 5.1.1. Detection and Recognition Effect

Good physical fitness is the foundation and guarantee of football, and it is also an important part of football practice teaching effect. This chapter will use the experimental group and the control group to carry out the experimental research of college football classroom practice teaching mode under big data. First, in order to test whether there is a difference in the physical fitness of the students, the students in the two classes were tested for 30 meters, 5*∗*25 reentry running, and standing long jump before the experiment began, and the two classes were premeasured with big data. Analyze the independent sample *T* test, and the results are as follows:

From Table 3, it can be concluded that the 30-meter score of the experimental group is 4.40 ± 0.08 s, the 5*∗*25 foldback running score is 33.79 ± 1.10 s, and the standing long jump is 2.67 ± 0.08 m; the 30-meter score of the control group is 4.37 ± 0.06, 5*∗*25 foldback running result was 33.09 ± 1.27 s, and the standing long jump was 2.70 ± 0.07 m. In order to see more clearly whether there are differences in the physical fitness of the two classes, an independent sample *T* test was performed on the results of the two classes, and the results showed that *P*_30 m_ = 0.083 > 0.05, *P*_Turn back_ = 0.072 > 0.05, *P*_standing long jump_ = 0.13 > 0.05. It can be seen that there is no difference in physical fitness between the experimental group and the control group, which meets the requirements of the experiment.

#### 5.1.2. Pretest Results and Analysis of Football Skills

In accordance with the test subject's college football assessment standards, the experimental group and the control group were tested on two indicators of ball bumping and shooting before the experiment. The two indicators are subjected to independent sample *T* test under big data analysis, and the results are as follows:

From Table 4 above, it can be concluded that the experimental group's smashing score was 2.35 ± 0.93 points, and the shooting score was 4.05 ± 0.95 points; the control group's smashing score was 2.75 ± 1.12 points, and the shooting score was 4.25 ± 1.21 points. Clearly see whether there is a difference in the physical fitness of the two classes. The results of the two classes are subjected to an independent sample *T* test under big data analysis, and the result is that *P*_Bumping the ball_ = 0.227 > 0.05, *P*_shot_ = 0.563 > 0.05, and it can be seen that there is no significant difference between the experimental group and the control group in the two techniques of throwing the ball and shooting. The football skills of the two classes are basically at the same level, which guarantees football teaching accuracy of teaching experiments.

Through the pretest, I have a basic understanding of the football skills of the students in the two classes. The test results reflect that the two classes have poor ball and shooting skills. Only a small number of students have good skills. According to the assessment standards, most students have a good performance. Being at a failing level, this shows that the students have not received professional and systematic football practice teaching before, and rarely do football sports after class.

### 5.2. The Results and Analysis of Each Index after the Experiment of the Experimental Group and the Control Group

After 16 weeks of continuous follow-up of practical teaching, the two classes of students' physical fitness, football technical level, and football theory learning three aspects were once again compared and analyzed. According to the syllabus of the subject colleges and universities, football skills accounted for 50%, football theory 30%, and football basic technology teaching 20% in the final assessment standards of football. For this reason, this article adds football theory and football basic technology teaching in the postexperimental test. Item test indicators were used in order to more comprehensively judge the impact of the college football classroom practice teaching system mode on the football teaching effect.

#### 5.2.1. Pretest Results and Analysis of Physical Fitness

*(1) Comparison of Physical Fitness between the Experimental Group and the Control Group before and after the Experiment*. In the last week of the experiment, the three indicators of the long jump were tested. The same as the previous test, the 30-meter run, 5*∗*25 reentry run, and standing of the two classes of students were tested to test whether the football practice teaching system model under big data has an impact on the physical fitness of students. The comparative data of the physical fitness of the experimental group and the control group before and after the experiment are as follows:

Table 5 shows that, after the experiment, the three indicators of the physical fitness of the experimental group before and after the experiment were, respectively, subjected to the paired sample *T* test under the big data analysis, and the result was *P*_30 m_ = 0.083 > 0.05, *P*_Turn back_ = 0.475 > 0.05, *P*_standing long jump_ = 0.087 > 0.05. It can be seen that, after the experiment, the physical fitness of the students in the experimental group does not change significantly, and there is no significant difference.

Table 6 shows that, after the experiment, the three indicators of the physical fitness of the control group before and after the experiment were, respectively, subjected to the paired sample *T* test under big data analysis, and the result was *P*_30 m_ = 0. 175 > 0.05, *P*_Turn back_ = 0.071 > 0.05, *P*_standing long jump_ = 0.385 > 0.05. It can be seen from this that, before and after the experiment, the three indicators of physical fitness of the control group students did not change significantly.

*(2) Comparison of Physical Fitness between the Experimental Group and the Control Group after the Experiment*.  Table 7 shows that, after the experiment, the three physical fitness scores of the two classes of students were subjected to an independent sample *T* test under big data analysis. The results showed that the *P* values of the three physical fitness items were all greater than 0.05. The experimental group and the control group were posttested. The physical fitness is not much different, and there is no significant difference.

**Figure 4:**
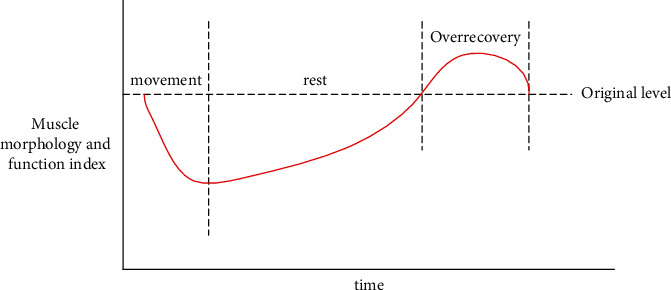
The law of human body function adaptability.

*(3) Analysis of Physical Fitness Test Results*. The above results show that, after the experiment, the physical fitness of the students in the experimental group has not significantly improved, and there is no significant difference between the physical fitness scores of the experimental group and the control group. The objective reasons above are mainly due to the following two points:

1. The human body is a gradual adaptation to sports stimulation, a cyclical process. Students' physical fitness and physical functions need to develop gradually and slowly from quantitative changes to qualitative changes in a continuous environment [22, 23]. In the 16-week college football classroom practice teaching under big data, if you want to improve the students' physical fitness, you must not only arrange the load intensity and load reasonably, but also follow the “law of human body function adaptability,” even if next time the load is arranged in the overload recovery stage of the last load, forming a relatively stable state of load-adaptation-additional load-readaptation (Figure 4) [24]. According to the schedule of football training sessions, the interval between the two sessions is too long. The load and recovery between classes cannot form an effective connection. Therefore, the physical fitness of students is difficult to significantly improve under various conditions.
2. From Tables 5 and 6, it can be seen that after, 16 weeks of football practical teaching under big data, the preexperimental results of the 5*∗*25 m reentry run of the experimental group and the control group were significantly better than the postexperimental results. The reason for the change of this indicator may be because the measured time after the experiment was December. The climate is cold in winter, and the human body will reflexively cause muscle and blood vessel contraction under cold stimulation, which will reduce the elasticity of muscles and ligaments, the joints are small, and the students' physical activity is not sufficient. The degree of excitement is not high, so the measured results can hardly reflect the true level of the students. The law of human body function adaptability has no essential causal relationship with data analysis, but it will have an impact on the results. In addition, the physical fitness of students is affected by physical conditions, environmental weather, and other factors. It is difficult to improve the physical fitness of students through this experiment. The focus of the football practice teaching system model under big data lies in cultivating students' autonomous learning ability and training result-oriented ability rather than improving students' physical fitness.

#### 5.2.2. Results and Analysis of the Posttest of Football Skills

*(1) The Comparison Result of Football Skill Level Test between the Experimental Group and the Control Group before and after the Experiment*. The level of football skills is an important indicator that reflects the implementation effect of the college football classroom practice teaching system model and the traditional classroom model under the big data. After 16 weeks of experimental observation, in order to test whether the college football classroom practice teaching system model under big data has a positive impact on students' football skills in football lessons, the experimental group and the control group football technical pre-and posttest scores are compared and analyzed with big data. The comparison results are as follows:

Tables 8 and 9 show that, through the paired-sample *T* test under the large number analysis of the scores of the experimental group and the control group before and after the experiment, the test results of the two classes after the experiment are higher than those of the experiment. The pretest results show that both the college football classroom practice teaching system model and the traditional teaching model under the big data can improve students' football skills.

*(2) The Comparison Result of Football Skill Level Test between the Experimental Group and the Control Group after the Experiment*. It can be seen from Table 10 that a comparative analysis of the scores of the ball and shooting of the experimental group and the control group shows that the two posttest scores of the experimental group are higher than those of the control group, and there is a very significant difference (*P* < 0.01).

*(3) Analysis of the Test Results of Football Skills*. According to the above results, after 16 weeks of practical teaching experiment observation, the football technical performance of the experimental group and the control group have improved, but the football technical level of the experimental group has improved more significantly. The analysis of the reasons is mainly in the following two aspects:

1. The comparative analysis of the pre- and posttest scores of the experimental group and the control group can be obtained: after 16 weeks of practical teaching experiment, under the big data college football classroom practical teaching system model and the traditional teaching model, the two classes of students level of ball and shooting skills have been significantly improved, which shows that the college football classroom practice teaching system model under big data still has a certain effect on the improvement of students' football skills, and the technical skill training for students is still very advantageous.
2. The comparative analysis of the football technical posttest scores of the experimental group and the control group can be obtained: after the experiment, the posttest scores of ball throws and shots of the experimental group are higher than those of the control group, and there are very significant differences. This shows that, in improving students' football skills, the implementation effect of the college football classroom practice teaching system model under big data is significantly better than the traditional classroom teaching model. Analyzed from the perspective of the law of formation of motor skills, students learn the teaching content pushed by teachers independently and correct training actions in time based on big data information feedback to form motor skills. The time for teachers to explain and demonstrate in class will be greatly shortened, and students will have enough time to reinforce exercises and practice. Teachers organize students' exercises and carry out differentiated error correction and personalized guidance through teachers' feedback and peer interaction, learning, discussing, abandoning the cognition of wrong movements, perfecting and optimizing one's own technical movements, so that the development of football skills can be improved through continuous feedback and correction. Due to the timely feedback of training data, students become more motivated to learn and have a stronger desire to achieve goals. Therefore, using the college football classroom practice teaching system model under big data for football teaching can improve the students' football skills, and its teaching effect is better than the traditional teaching model.

#### 5.2.3. The Results and Analysis of the Posttest of Football Theoretical Results

*(1) The Comparison Result of the Experimental Group and the Control Group after the Experiment of Football Theory Score Test*. Football projects focus on the combination of theory and practice, and mastering theoretical knowledge can greatly promote the learning of football skills. Therefore, the degree of students' mastery of theory is a key indicator reflecting the effectiveness of college football classroom practice teaching under big data. After the 16-week experimental observation, a posttest comparative analysis of the theoretical football scores of the experimental group and the control group was carried out. The results are as follows:

As can be seen from the above table, after the experiment, the theoretical scores of the two classes of students are processed by independent sample *T* test under big data. The results show that the theoretical scores of the experimental group are 88.10 ± 4.58 points, and the theoretical scores of the control group are 76.15 ± 8.60 points. *P* < 0.01, the theoretical posttest scores of the experimental group are significantly higher than those of the control group, and there are very obvious differences.

*(2) Analysis of the Test Results of Football Theory*. It can be seen from Table 11 that, after the experiment, there is a significant difference between the experimental group and the control group in football theoretical performance, indicating that college football classroom practice teaching under big data is more conducive to improving students' grasp of football theoretical knowledge than traditional teaching. A detailed analysis of the reason is mainly due to the fact that college football classroom practice teaching under big data is goal- and result-oriented, programmatic learning and effective feedback can be carried out, and the feedback of big data information can be used for timely evaluation and further strengthening of learning during the learning process. In addition, in the classroom, the teacher can use the results of the systematic evaluation and recording to further intervene in the learning situation of the students. In order to improve the level of football skills in the learning and practice, the students will actively think about the technical movements and strengthen the theory of football while mastering football skills, understanding and deepening of knowledge.

## 6. Conclusion

This paper studies the college football classroom practice teaching system model based on big data analysis and carries out a procedural design for the college football classroom teaching process. Under the big data analysis, the corresponding evaluation indicators are selected according to the weight of the indicators to carry out experimental comparative research. According to the continuous collection of data in the teaching practice, the following conclusions are drawn: (1) the experimental group and the control group under the big data analysis have little difference in physical fitness after testing, and there is no significant difference. (2) Fully adopting the college football classroom practice teaching system model under big data to carry out football teaching can improve the football skills of students, and its teaching effect is better than traditional teaching mode. (3) College football classroom practice teaching under big data focuses on goal and result orientation and can carry out procedural learning and effective feedback. In the learning process, the feedback of big data system information can be evaluated in time and further strengthened to strengthen the theory.

## Figures and Tables

**Table 1:** Quantitative screening scores of physical fitness test indicators.

Physical fitness test index	Influence level					
	Very important 5 points	Important 4 points	Generally 3 points	Not so important 2 points	Unimportant 1 points	Total score
30-meter sprint	4	2	0	0	0	28
12 minutes run	1	1	0	2	2	15
Standing long jump	3	2	1	0	0	26
YOYO	1	2	0	3	0	19
1000 m	0	0	3	2	1	14
Back and forth	5	1	0	0	0	29

Note. Perform linear interpolation scoring based on the quantitative results.

**Table 2:** Score and score calculation table.

Score	Male	3	4	5	6	8	10	15	20	25
	Female	2	3	4	5	6	8	10	15	20

Points		2	3	4	5	6	7	8	9	10

**Table 3:** Premeasurement results of physical fitness of the experimental group and the control group.

Test index	Test group ($\bar{X}\pm S$) (*N* = 20)	Control group ($\bar{X}\pm S$) (*N* = 20)	*T* value	*P* value
30 m (s)	4.40 ± 0.08	4.37 ± 0.06	1.782	0.083
Turn back (s)	33.79 ± 1.10	33.09 ± 1.27	1.848	0.072
Standing long jump (m)	2.67 ± 0.08	2.70 ± 0.07	−1.547	0.130

**Table 4:** Premeasurement results of football skills of the experimental group and the control group.

Test content	Test group ($\bar{X}\pm S$) (*N* = 20)	Control group ($\bar{X}\pm S$) (*N* = 20)	*T* value	*P* value
Bumping the ball (points)	2.35 ± 0.93	2.75 ± 1.12	−1.228	0.227
Shots (points)	4.05 ± 0.95	4.25 ± 1.21	−0.583	0.563

**Table 5:** Comparison and quantitative results of the physical fitness of the experimental group before and after the experiment.

Test index	Before the experiment ($\bar{X}\pm S$) (*N* = 20)	After the experiment ($\bar{X}\pm S$) (*N* = 20)	*T* value	*P* value
30 m (s)	4.40 ± 0.08	4.38 ± 0.05	1.833	0.083
Turn back (s)	33.79 ± 1.10	34.02 ± 0.93	−0.728	0.475
Standing long jump (m)	2.67 ± 0.08	2.68 ± 0.07	−1.807	0.087

**Table 6:** Comparative quantitative results of physical fitness before and after the experiment of the control group.

Test index	Before the experiment ($\bar{X}\pm S$) (*N* = 20)	After the experiment ($\bar{X}\pm S$) (*N* = 20)	*T* value	*P* value
30 m (s)	4.37 ± 0.06	4.38 ± 0.05	−1.407	0.175
Turn back (s)	33.09 ± 1.27	33.58 ± 0.74	−1.916	0.071
Standing long jump (m)	2.70 ± 0.07	2.71 ± 0.07	−0.890	0.385

**Table 7:** Quantified comparison results of physical fitness between the experimental group and the control group after the experiment.

Test index	Before the experiment ($\bar{X}\pm S$) (*N* = 20)	After the experiment ($\bar{X}\pm S$) (*N* = 20)	*T* value	*P* value
30 m (s)	4.38 ± 0.05	4.38 ± 0.05	−0.271	0.788
Turn back (s)	34.02 ± 0.93	33.58 ± 0.74	1.656	0.106
Standing long jump (m)	2.68 ± 0.07	2.71 ± 0.07	−1.394	0.107

**Table 8:** Results of quantitative comparison of football skills before and after the experiment in the experimental group.

Test index	Before the experiment ($\bar{X}\pm S$) (*N* = 20)	After the experiment ($\bar{X}\pm S$) (*N* = 20)	*T* value	*P* value
Bumping the ball (points)	2.35 ± 0.93	6.95 ± 1.73	−13.707	*P* < 0.001
Shots (points)	4.05 ± 0.95	7.50 ± 1.24	−11.716	*P* < 0.001

**Table 9:** Quantitative comparison results of football skills before and after the experiment of the control group.

Test index	Before the experiment ($\bar{X}\pm S$) (*N* = 20)	After the experiment ($\bar{X}\pm S$) (*N* = 20)	*T* value	*P* value
Bumping the ball (points)	2.75 ± 1.12	5.25 ± 1.02	−11.180	*P* < 0.001
Shots (points)	4.25 ± 1.21	6.25 ± 1.12	−6.325	*P* < 0.001

**Table 10:** Quantified comparison results of football skills between the experimental group and the control group after the experiment.

Test index	Before the experiment ($\bar{X}\pm S$) (*N* = 20)	After the experiment ($\bar{X}\pm S$) (*N* = 20)	*T* value	*P* value
Bumping the ball (points)	6.95 ± 1.73	5.25 ± 1.02	3.784	*P* < 0.001
Shots (points)	7.50 ± 1.24	6.25 ± 1.12	3.355	*P* < 0.001

**Table 11:** Comparison results of theoretical football scores between the experimental group and the control group after the experiment.

Content	Test group ($\bar{X}\pm S$) (*N* = 20)	Control group ($\bar{X}\pm S$) (*N* = 20)	*T* value	*P* value
Football theory score (100 points)	88.10 ± 4.58	76.15 ± 8.60	5.484	*P* < 0.001

## Data Availability

The dataset can be accessed upon request.
